# Prevalence of liver fibrosis and risk factors in a general population using non-invasive biomarkers (FibroTest)

**DOI:** 10.1186/1471-230X-10-40

**Published:** 2010-04-22

**Authors:** Thierry Poynard, Pascal Lebray, Patrick Ingiliz, Anne Varaut, Brigitte Varsat, Yen Ngo, Pascal Norha, Mona Munteanu, Fabienne Drane, Djamila Messous, Françoise Imbert Bismut, Jean Pierre Carrau, Julien Massard, Vlad Ratziu, Jean Pierre Giordanella

**Affiliations:** 1 APHP-UPMC-Liver Center, Assistance Publique Hôpitaux de Paris, Université Pierre et Marie Curie, Paris, France; 2 Caisse Primaire Assurance Maladie, Paris, France; 3 Biopredictive, Paris, France; 4 Biochemistry Unit, Assistance Publique Hôpitaux de Paris, Université Pierre et Marie Curie, Paris, France

## Abstract

**Background:**

FibroTest and elastography have been validated as biomarkers of liver fibrosis in the most frequent chronic liver diseases and in the fibrosis screening of patients with diabetes. One challenge was to use them for estimating the prevalence of fibrosis, identifying independent risk factors and to propose screening strategies in the general population.

**Methods:**

We prospectively studied 7,463 consecutive subjects aged 40 years or older. Subjects with presumed advanced fibrosis (FibroTest greater than 0.48) were re-investigated in a tertiary center.

**Results:**

The sample characteristics were similar to those of the French population. FibroTest was interpretable in 99.6%. The prevalence of presumed fibrosis was 2.8%, (209/7,463), including cirrhosis in 0.3% (25/7,463); 105/209 (50%) subjects with presumed fibrosis accepted re-investigation. Fibrosis was confirmed in 50, still suspected in 27, indeterminate in 25 and not confirmed with false positive FibroTest or false negative elastography in 3 subjects. False negative rate of FibroTest estimated using elastography was 0.4% (3/766). The attributable causes for confirmed fibrosis were both alcoholic and nonalcoholic fatty liver disease (NAFLD) in 66%, NAFLD in 13%, alcohol in 9%, HCV in 6%, and other in 6%. Factors independently associated (all P < 0.003) with confirmed fibrosis were age, male gender, waist circumference, HCV antibody and alcohol consumption estimated using carbohydrate-deficient transferrin, enabling efficient screening-oriented strategies to be compared and proposed.

**Conclusions:**

Biomarkers have permitted to estimate prevalence of advanced fibrosis around 2.8% in a general population aged 40 years or older, and several risk factors which may be used for the validation of selective or non-selective screening strategies.

## Background

Screening for liver fibrosis is increasingly appropriate as three major recommended criteria have now been fulfilled, including the disease severity, the tests accuracy and the effectiveness of treatments [1].

First, liver fibrosis is a significant health problem with a worldwide mortality attributable to cirrhosis and primary liver cancer of around 1.5 millions death per year, with in France (1/100 of world population) a similar mortality rate around 15,000 death per year [2]. Cirrhosis is the last stage of fibrosis which occurs mainly in response to viral and toxic-metabolic insults. The most common causes of fibrosis progression are chronic hepatitis C, chronic hepatitis B, alcoholic liver disease and nonalcoholic fatty liver disease [3].

Second, liver fibrosis is detectable with non-invasive markers [4-6]. They are many fibrosis biomarkers and the two most investigated and validated biomarkers are the FibroTest (FT), a serum in vitro multivariate assay (FibroTest^®^, Biopredictive, Paris, France; FibroSURE^® ^in the USA, LabCorp, Burlington), and the Fibroscan [Echosens, Paris, France], a measure of liver stiffness (LSM) using elastography [4-9]. FT and LSM have similar accuracy for the diagnosis of cirrhosis, with FT having higher sensitivity for the diagnosis of earlier stages of fibrosis[10]. FT has already been evaluated in two screening studies in high-risk populations: a retrospective study in hyperlipidemic subjects and a prospective study in diabetic subjects. These results were concordant with a prevalence of presumed advanced (bridging) fibrosis of 6% in subjects with type 2 diabetes [11,12].

Third, liver fibrosis is treatable, even at the cirrhotic stage, mainly using anti-viral treatments for hepatitis C and B, but also by reducing alcohol consumption and improving overweight, diabetes and metabolic factors for nonalcoholic fatty liver disease [13]. Whatever the cause of cirrhosis, prevention of variceal bleeding and detection of small liver cancer in patients with cirrhosis can improve survival [12,14].

The aim of the present pilot study was to assess the feasibility of the first steps of fibrosis screening using biomarkers. Screening is a chain of activities that starts with defining the target population and extends to the treatment and follow-up of the screen-detected patients.^1 ^The aim was not to re-assess biomarkers' accuracy using biopsy as this mandatory step has been extensively validated in the most frequent chronic liver diseases including methods without perfect gold standards [10].

The primary aim was to use FT, as a first-line sensitive test and elastography as a confirmation test to estimate the prevalence of advanced fibrosis in a general population. The secondary aim was to identify independent risk factors of fibrosis and to compare the accuracy of a non selective screening versus selective screenings.

## Methods

### Inclusion criteria

Consecutive subjects, forty years of age or older, who were seen for a free screening program in two French Social Security health examination centers were eligible for inclusion. All procedures were performed in accordance with the current revised guidelines of the Declaration of Helsinki, approved by the ethical committee of Groupe Hospitalier Pitié Salpêtrière and all investigated participants gave informed signed consent. Forty years threshold was chosen as fibrosis progression is very slow in younger patients [3].

### Initial medical screening

Screened subjects filled out a questionnaire, underwent an interview and physical examination by a physician, and blood sampling. The questionnaire included 70 epidemiological, clinical and environmental characteristics. The blood sample included liver function tests, fasting glucose, lipids, and biomarkers of fibrosis (FT), steatosis (SteatoTest), and NASH (NashTest) [5,6] (Table 1). In a consecutive subpopulation the serum carbohydrate-deficient transferrin (CDT) was measured in order to improve the identification of subjects with excessive drinking [15].

**Table 1:** Characteristics of naive subjects with and without presumed advanced fibrosis

Characteristics	Subjects with presumed fibrosis^1 ^reinvestigated	Subjects with presumed fibrosis^1 ^not reinvestigated	Subjects without presumed fibrosis
Number of subjects	105	104	7254
Age at serum, years (mean;95%CI)	66.5 (64.7-68.2)	63.6 (62.0-65.2)	56.7 (56.5-56.9)
Male (%; 95%CI)	94 (0.90;0.82-0.95)	95 (0.91;0.84-0.96)	3924 (0.54;0.53-0.55)
Tobacco consumption	63/105 (0.60;0.50-0.69)	54/104 (0.52;0.42-0.62)	3245/7247 (0.45;0.44-0.46)
No physical exercise	65/105 (0.62;0.52-0.71)	60/104 (0.58;0.48-0.67)	4685/7247 (0.65;0.64-0.66)
Retired	70 (0.67;0.57-0.76)	58 (0.56;0.46-0.66)	2224 (0.31;0.30-0.32)
**Fatty liver risk factor (Alcohol or metabolic)**	**98 (0.93;0.87-0.97)**	**86 (0.83;0.74-0.89)**	**4698 (0.65;0.64-0.66)**
Mean daily self-reported alcohol consumption	11.6 (8.2-15.0)	15.3 (11.4-19.3)	9.9 (9.6-10.3)
Self-reported alcohol consumption at risk^2^	24/105 (0.23;0.15-0.32)	28/104 (0.27;0.19-0.37)	1634/7247 (0.23;0.22-0.24)
CDT assessed	72/105 (0.69;0.59-0.77)	2/104 (0.02;0.00-0.07)	1023/7254 (0.14;0.13-0.15)
Carbohydrate Deficient Transferin	1.92 (1.76-2.07)	1.49 (1.24-1.74)	1.51 (1.48-1.54)
Elevated Carbohydrate Deficient Transferin (>1.6%)^3^	45/72 (0.63;0.50-0.74)	NP	303/1023 (0.30;0.27-0.33)
Alcohol at risk (either reported consumption or CDT)^3^	54/72 (0.75;0.63-0.85)	NP	426/1023 (42%)
BMI >= 27.0	57 (0.54;0.44-0.64)	49 (0.47;0.37-0.57)	2319/7245 (0.32;0.31-0.33)
***Metabolic factor of ATP-III classification (at least one)***	***83 (0.79;0.70-0.86)***	***80 (0.77;0.68-0.85)***	***3827 (0.53;0.52-0.54)***
Glucose >= 6.1 mmol/L or diabetes treatment	34/103 (0.33;0.24-0.43)	42/104 (0.40;0.31-0.51)	1069/7253 (0.15;0.14-0.16)
Central obesity waist >102 male >88 female	30/105 (0.29;0.20-0.38)	28/104 (0.27;0.19-0.37)	1162/7245 (0.16;0.15-0.17)
Triglycerides >= 1.7 mmol/L or fibrate treatment	43/101 (0.41;0.32-0.51)	37/104 (0.36;0.26-0.46)	1707/7199 (0.24;0.23-0.25)
Hypertension or treatment	44/105 (0.42;0.32-0.52)	41/104 (0.39;0.30-0.50)	1885/7240 (0.26;0.25-0.27)
HDL-cholesterol <1.03 mmol/L male <1.29 mmol/L female missing 2564	16/72 (0.22;0.13-0.34)	12/68 (0.18; 0.10-0.29)	328/4764 (0.07;0.06-0.08)
**Steatosis predicted by SteatoTest**			
No	23/101 (0.23;0.15-0.32)	31/104 (0.30;0.21-0.40)	4174/7190 (0.58;0.57-0.59)
Minimal (1-5%)	37/101 (0.37;0.27-0.47)	33/104 (0.32;0.23-0.42)	1617/7190 (0.23;0.22-0.24)
Moderate (6-33)	19/101 (0.19;0.12-0.28)	15/104 (0.14;0.08-0.23)	706/7190 (0.10;0.09-0.11)
Marked-Severe (34-100%)	22/101 (0.22;0.14-0.31)	25/104 (0.24;0.16-0.33)	693/7190 (0.10;0.09-0.10)
Steatohepatitis predicted by NASHTest	7/101 (0.07;0.03-0.13)	11/104 (0.11;0.05-0.18)	62/7190 (0.009;0.007-0.011)
Risk HCV (Transfusion, tattoo, piercing, heroin, cocaine)	1 (0.010;0.000-0.052)	1 (0.010;0.000-0.052)	163 (0.023;0.019-0.026)
HCV antibody assessed	105/105 (1.00;0.97-1.00)	52/104 (0.50;0.40-0.60)	3473/7254 (0.48;0.47;0.49)
HCV antibody positive	5/105 (0.05;0.02-0.11)	1/52 (0.02;0.00-0.10)	26/3473 (0.008;0.005-0.011)
HIV antibody positive	1/63 (0.02;0.00-0.09)	0/6 (0.00;0.00-0.46)	1/944 (0.001;0.000-0.006)
HBsAg assessed	105/105 (1.00;0.97-1.00)	8/104 (0.08;0.03-015)	604/7254 (0.08;0.077-0.090)
HBsAg antigen positive	2/105 (0.02;0.00-0.07)	0/104 (0.00;0.00-0.04)	5/604 (0.008; 0.003-0.019)
Liver stiffness measurement assessed	93/105 (0.86;0.81-0.94)	0/104 (0.00;0.00-0.04)	865/7254 (0.12;0.11-0.13)
LSM kPa	10.1 (8.3-12.0)	NP	5.0 (4.8-5.1)
**Markers (normal range)**			
AST IU/L (17-27 female; 20-32 male)	34 (31-37)	40 (34-46)	24 (24-24)
ALT IU/L (11-26 female; 16-35 male)	43 (37-50)	46 (38-54)	27 (26-27)
Total bilirubin mol/L (1-21)	16 (14-17)	16 (14-17)	12 (12-12)
GGT U/L (7-32 female; 11-49 male)	72 (56-87)	111 (75-146)	27 (26-27)
Alpha 2 macroglobulin g/L (female 1·6-4·0; male 1·4-3·3)	2.4 (2.3-2.5)	2.4 (2.3-2.5)	1.5 (1.5-1.5)
Apo A1 g/L (1·2-1·7)	1.5 (1.4-1.5)	1.5 (1.4-1.5)	1.7 (1.7-1.7)
Haptoglobin g/L (0·35-2·00)	0.9 (0.8-1.0)	0.8 (0.7-0.9)	1.2 (1.1-1.2)
Fasting Glucose (mmol/L)	6.1 (5.8-6.4)	6.4 (6.0-6.8)	5.5 (5.5-5.5)
Cholesterol (mmol/L)	5.3 (5.1-5.5)	5.4 (5.1-5.6)	5.8 (5.7-5.8)
Triglycerides (mmol/L)	1.7 (1.3-2.0)	1.8 (1.4-2.2)	1.3 (1.3-1.3)
HDL-cholesterol (data in 72/68/4764 subjects)	0.49 (0.46-0.51)	0.51 (0.47-0.54)	0.63 (0.62-0.63)

^1 ^FibroTest greater than 0.48

^2 ^More than one drink per day for females and more than two drinks per day for males

Data are mean (SD) or proportion.

^3 ^Carbohydrate Deficient Transferin was consecutively assessed in 1038 subjects at baseline and in 59 subjects during reinvestigations.

The previous standard of care in these prevention centers remained unchanged. There was already a "viral hepatitis C or B"-oriented strategy. When the physician of the prevention center suspected a risk of HCV or HBV infection or if the transaminase ALT level was above normal, an HCV antibody or HBsAg antigen assay was routinely performed and the patient was re-investigated if they were positive.

### Reinvestigation

Advanced fibrosis was "presumed" when FT was greater than 0.48. This threshold was validated for METAVIR scoring system advanced fibrosis (few septa, many septa, cirrhosis) in HCV, HBV and equivalent bridging fibrosis in ALD and NAFLD [3]. Subjects with presumed fibrosis were contacted for a reinvestigation in the reference center. Reinvestigation consisted of a hepatologist consultation, a second FT, an elastography (LSM), serum markers of chronic liver disease, and if necessary, liver ultrasonography, esophageal endoscopy, or liver biopsy.

### Analysis Design

The primary analysis estimated the prevalence of fibrosis and its associated risk factors and was performed in all included subjects without any previous history of liver disease.

The secondary analysis compared the non selective screening to three risk-oriented strategies. The HCV/HBV-oriented risk population was the previous standard of care. The alcohol-oriented risk population included all subjects who drank more than 10 grams a day for females and more than 20 grams for males [16]. The metabolic syndrome-oriented population included all subjects with at least one factor of the metabolic syndrome (Table 1).

A third analysis was planned to assess the number of possible false negative cases missed using FT alone. In a consecutive population, FT and LSM were performed on the same day in the prevention center by the same operators of the reference center.

### Sample size

For the primary analysis it had been estimated that at least 7,500 subjects would be needed in order to reinvestigate at least 100 subjects in the reference center, assuming a prevalence of 3% and an acceptance rate of 50%.

### Biomarkers measurements

FT includes α_2_-macroglobulin, apolipoprotein A1, haptoglobin, total bilirubin, and γ-glutamyl-transpeptidase (GGT), adjusted for age and gender. FT scores range from zero to 1.00. The FT, SteatoTest, NashTest components were analyzed according to published recommendations [5,6].

LSM is expressed in kilopascals (kPa). The technique was performed by experienced hepatologists who were blinded, and was done according to the manufacturer's recommendations [10,17,18]. The predefined threshold for advanced fibrosis was 7.1 kPa, 12.5 kPa for cirrhosis and 5 kPa for minimal fibrosis [10,17-19].

### Endpoints

All cutoffs were defined a priori. Advanced fibrosis was "confirmed" if FT was greater than 0.48 and elastography was 7.1 kPa or above; or there were endoscopic signs of portal hypertension; or advanced fibrosis was demonstrated on liver biopsy. Advanced fibrosis was "still suspected" if FT was greater than 0.48 and LSM was between 5.0 kPa and 7.1 kPa as LSM has a lower sensitivity than FT.^10 ^Fibrosis was "indeterminate with suspected false positive FT or false negative LSM" if FT was greater than 0.48 and the LSM was lower than 5.0 kPa.

The efficacy of the risk-oriented strategies was compared using the number of identified advanced fibrosis cases, the relative risk of advanced fibrosis as estimated by the odds ratio, the AUROC for quantitative factors and the number of persons required to undergo fibrosis screening in order to detect one case of advanced fibrosis.

In the subpopulation with CDT measurement, the goal was to assess whether CDT was a better predictive factor than self-reported alcohol consumption for the prediction of advanced fibrosis and for attributing the cause of liver disease.

In the subpopulation that was also screened with LSM, the goal was to estimate the percentage of FT false negatives. Subjects with LSM 7.1 kPa or greater were systematically contacted for reinvestigation.

### Statistical analysis

The Fisher's exact, Mann-Whitney, Bonferroni, and Tukey-Kramer tests and logistic regression were used. For diagnostic values, relative risk was estimated using the odds ratio. The area under the ROC curve (AUROC) was assessed for quantitative factors. The representativeness of the present general population sample enrolled in the French Social Security health examination center sample was assessed using comparisons with characteristics of the overall French population and from a French national survey on nutrition and health [20,21].

Number Cruncher Statistical Systems 2007 software (NCSS, Kaysville, Utah, USA) was used [22].

## Results

### Screened population

Between June 2006 and September 2008, 7,554 subjects were eligible; 72 were excluded, including 33 because of high-risk profiles of FT false positives or false negatives (applicability 99.6%; 7521/7554), and 16 protocol refusal (acceptance 99.8%; 7505/7521). Majority of high-risk profiles (24/33) were abnormally low haptoglobin (hemolysis or anhaptoglobinemia), 4 subjects had abnormally high apoA1 value, 1 abnormally low ApoA1, 2 abnormally high GGT, one Gilbert syndrome with 72 micromol of unconjugated bilirubin, and one abnormally high A2 M.

**Figure 1:**
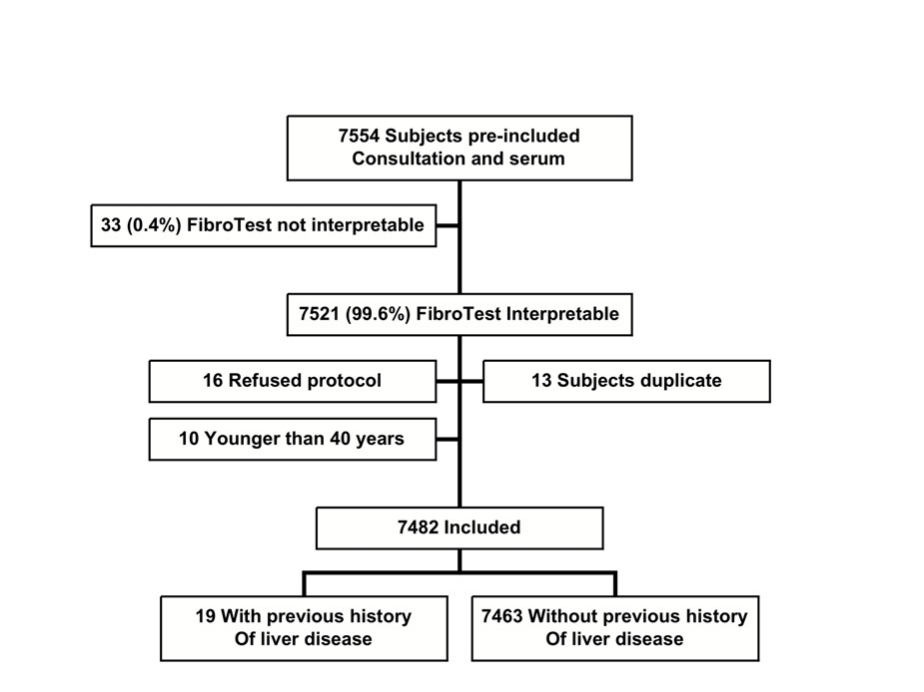
**Flow sheet of subjects included**.

Among the 7,482 included subjects, 7,463 had no history of liver disease (naive population) and 19 had a history (Figure 1). The characteristics of the included population were similar to those of the French general population (Additional file 1) [21,22]. A total of 3362/7482 (45%) subjects received at least one treatment the day of inclusion, but no specific details were available.

### Prevalence of fibrosis

In the naïve population, 209/7463 (2.8%; 2.4%-3.2%) subjects [N (%:95%CI)] had a FT with presumed fibrosis and 25 with presumed cirrhosis (0.3%; 0.2%-0.5%); 1336/7395 (18.1%; 17.2%-18.9%) had a SteatoTest with presumed steatosis (over 5% of hepatocytes) and 80/7463 (1.1%; 0.8%-1.3%) had a NashTest with presumed steato-hepatitis.

A total of 105 subjects accepted to be reinvestigated (50% adherence) and were similar to the 104 subjects that were not reinvestigated (Table 1). Fibrosis was confirmed in 50, still suspected in 27, and indeterminate with a suspected false positive of FT or false negative of LSM in 28 (Table 2). Only four subjects (4%) accepted liver biopsy out of the 105 that were reinvestigated. Nine subjects had confirmed cirrhosis, all without any clinically obvious signs. One subject had small esophageal varices. Non-alcoholic and alcoholic fatty liver diseases were the most frequent etiologies (85%) for advanced fibrosis (Table 2). Among patients with confirmed cirrhosis, 22% had HCV (all associated with ALD), 44% had ALD and NAFLD and 33% had NAFLD alone (Additional File 2). Correlation between first and second FT was 0.77 (P < 0.0001) with a significant concordance between cirrhosis/non cirrhosis (kappa = 0.76; P < 0.001).

**Table 2:** Characteristics of 209 subjects with FibroTest >0.48 (presumed advanced fibrosis) in the population without a history of liver disease

Characteristics	All	Reinvestigated				Not reinvestigated
	**Presumed fibrosis**	**Fibrosis Confirmed**	**Fibrosis still suspected**	**Indeterminate**	**All reinvestigated**	
Number of subjects	209	50	27	28	105	104
Prevalence of fibrosis^1^	209/7,463 (2.8%)	100/7,463 (1.3%)	54/7,463 (0.7%)	56/7,463 (0.8%)	100/7,463 (1.4%)	104/7,463 (1.4%)
Cause of liver disease						
Non alcoholic fatty liver disease^2^	98 (47%)	18 (35%)	10 (40%)	13 (46%)	41 (39%)	57 (55%)
Alcoholic liver disease^3^	15 (7%)	4 (8%)	1 (4%)	4 (14%)	9 (9%)	6 (6%)
Non alcoholic and alcoholic	61 (29%)	22 (42%)	11 (44%)	6 (21%)	39 (37%)	22 (21%)
Chronic hepatitis C	6 (3.5%)	4 (8%)	0	1 (4%)	5 (5%)	1 (1%)
Chronic hepatitis B	3 (1.4%)	1 (2%)	1 (4%)	0	3 (3%)	0 (0%)
Hemochromatosis	1 (0.5%)	1 (2%)	0	0	1 (1%)	0 (0%)
Auto-immune hepatitis	1 (0.5%)	0 (0%)	1 (4%)	0	1 (1%)	0 (0%)
No risk factor	25 (12%)	2 (4%)	1 (4%)	3 (11%)	7 (7%)	18 (17%)
Liver complications						
Hepatocellular carcinoma	NP	0 (0%)	0	0	0	NP
Portal hypertension	NP	1 (2%)	0	0	1 (1%)	NP
Stage presumed fibrosis						
Few septa	128 (61%)	26 (50%)	18 (72%)	22 (79%)	66 (63%)	62 (60%)
Many septa	56 (27%)	17 (33%)	6 (24%)	5 (18%)	28 (27%)	28 (27%)
Cirrhosis	25 (12%)	9 (17%)	1 (4%)	1 (3%)	11 (10%)	14 (13%)
Mode of confirmation^4^						
Elastography	NP	47 (90%) (>= 7.1 kPa)	27 (100%) (5kPa-7kPa)	28 (100%) (<5kPa)	102(95%)	NP
Biopsy	NP	3 (5%)	0	1 (4%)	4 (4%)	NP
Endoscopy	NP	1 (2%)	0	0	1 (1%)	NP

^1 ^Estimated prevalence assuming that the prevalence of advanced ?brosis would be the same in the population of patients not reinvestigated. ^2^At least one factor of the metabolic syndrome without alcohol consumption at risk ^3^Alcohol consumption at risk self-reported or CDT >1.6% without metabolic factor ^4^Possibility of several confirmations for the same subject NP = not performed

The estimated prevalence of confirmed fibrosis was 1.3% (1.1%-1.7%) (Table 2). In a "worse" scenario assuming that fibrosis was not present in patients not reinvestigated, these overall estimated prevalences would have been 0.7%.

Among the 19 subjects of the non-naïve population, 16 (84%) had an FT with presumed fibrosis; 13 subjects were reinvestigated and the presumed fibrosis was confirmed in 12 (positive predictive value 92%) using 10 LSM and seven biopsies. If included, the prevalence of advanced fibrosis ranged from 1.5% [(100+12)/(7463+19)] for confirmed fibrosis to 3% [(209+16)/7463+19)] for presumed fibrosis.

### Factors associated with fibrosis

In univariate (Table 3) and multivariate analyses (Table 4), five factors were consistently and independently associated with fibrosis (confirmed, or presumed): age, male gender, waist circumference, alcohol consumption estimated using CDT, and positive HCV antibodies. Reported alcohol consumption was not associated with advanced fibrosis. Serum triglycerides were no longer associated with fibrosis when CDT was included in the model (Table 4).

**Table 3:** Predictive values of oriented screening strategies

Strategy	Number subjects	Presumed fibrosis	Confirmed fibrosis
*Prevalence fibrosis*^1^	*7463*	*209 (2.8%)*	*50 (0.7-1.4%)*
**Metabolic factors oriented**			
Predictive value			
At least one metabolic factor	3990	163 (4.1%)	40 (1.0%)
None	3473	46 (1.3%)	10 (0.3%)
Odds ratio	7463	3.2 (2.3-4.5)	3.4 (1.7-7.5)
Area under ROC curve	4854	0.69(0.64-0.73)	0.71 (0.59-0.80)
**Alcohol-oriented per self-**			
***reported consumption***			
Predictive value			
>10 g female/20 g male	1686	52 (3.1%)	15 (0.9%)
<= 10 g female/<= 20 g male	5770	157 (2.7%)	35 (0.6%)
Odds Ratio	7456	1.1 (0.8-1.6)	1.5 (0.8-2.8)
Area under ROC curve	7456	0.55(0.51-0.59)	0.52 (0.43-0.60)
***CDT oriented***			
Predictive value			
CDT>1.6	348	45 (12.9%)	22 (6.3%)
CDT<= 1.6	749	29 (3.9%)	8 (1.1%)
Odds Ratio	1097	3.7 (2.2-6.2)	6.1 (2.6-15.4)
Area under ROC curve	1097	0.72(0.64-0.78)	0.75 (0.67-0.86)
**Hepatitis Virus-oriented**^2^			
Predictive value			
HBsAg or HCV antibody	36	5 (13.9%)	3 (8.3%)
HBsAg and HCV negative or not done at baseline	7427	204 (2.7%)	47 (0.6%)
Odds ratio	7463	5.9 (1.9-15.6)	15.3 (3.4-50.9)
**Transaminases- oriented**			
Predictive value			
ALT >= 50 IU/L	513	53 (10.3%)	17 (3.3%)
ALT < 50 IU/L	6950	156 (2.2%)	33 (0.5%)
Odds ratio	7463	5.0 (3.6-7.0)	7.2 (3.8-13.4)
Area under ROC curve	7463	0.72(0.68-0.75)	0.78 (0.72-0.83)
***Age-oriented***			
Predictive value			
Age > 60 years	2960	156 (5.3%)	42 (1.4%)
Age <= 60 years	4503	53 (1.2%)	8 (0.2%)
Odds ratio	7463	4.7 (3.4-6.5)	8.3 (3.8-19.5)
Area under ROC curve	7463	0.75(0.72-0.78)	0.79 (0.71-0.84)
***Gender-oriented***			
Male	4113	189 (4.6%)	47 (1.1%)
Female	3350	20 (0.6%)	3 (0.1%)
Odds ratio	7463	8.0 (5.0-13.1)	12.0 (4.0-54.1)

^1 ^Upper estimated prevalence assuming that the prevalence of advanced fibrosis would be the same in the population of patients not reinvestigated. Lower prevalence assuming that no advanced fibrosis was present among patients not reinvestigated.

^2 ^This strategy was the standard in the screening centers. Two cases with positive HBsAg detected during reinvestigations of advanced fibrosis were not taken into account. There was one coinfection HCV-HBV.

**Table 4:** Multivariate analysis of factor associated with advanced fibrosis

Factor	**Presumed fibrosis**^1^	**Confirmed fibrosis**^1^
		*OR (95%CI)*
**All subjects n = 7395**	**R2 = 0.22**	**R2 = 0.20**
Age	1.12 (1.10-1.14) <0.0001	1.13 (1.09-1.16) <0.0001
Male gender	4.31 (2.62-7.08) <0.0001	6.36 (2.03-22.1) 0.002
Waist	1.03 (1.01-1.04) 0.0002	1.05 (1.02-1.07) 0.001
Triglycerides^1^	1.32 (1.21-1.45) <0.0001	1.22 (1.07-1.39) 0.001
Total cholesterol	0.61 (0.52-0.72) 0.04	0.81 (0.60-1.11) 0.19
Fasting glucose	1.15 (1.05-1.26) 0.002	1.11 (0.94-1.32) 0.21
Reported alcohol	1.00 (0.99-1.01)	0.99 (0.97-1.01) 0.33
**CDT assays^1 ^n = 1076**	**R2 = 0.31**	**R2 = 0.31**
CDT	2.09 (1.42-3.05) 0.0002	2.11 (1.39-3.20) 0.0005
**CDT assays and HCV antibody^1 ^testing n = 500**	**R2 = 0.35**	**R2 = 0.36**
CDT	1.82 (1.21-2.73) 0.004	1.78 (1.24-2.56) 0.01
HCV positive	18.0 (2.43-132.7) 0.005	26.8 (2.75-261.3) 0.005

^1 ^When CDT entered the models, the impact of serum triglycerides disappeared.

### Comparison of screening strategies efficacy (Table 3)

The non selective strategy using FT in all subjects identified 0.7% (50/7,463) confirmed fibrosis cases, i.e., 149 subjects would be required to undergo fibrosis screening in order to detect one case of advanced fibrosis.

The most sensitive selective screening strategy was the metabolic factor-oriented strategy, which identified 40/50 (80%) confirmed advanced fibrosis cases, significantly more than the alcohol-oriented strategy using self-reported alcohol consumption and significantly more than the standard HCV/HBV-oriented strategy.

Among 1097 subjects who had CDT measurements (Additional File 3), the alcohol-oriented strategy using CDT identified 22/30 (73%) confirmed fibrosis cases, which did not differ from the metabolic strategy (23/30, 77%; P = 0.78 versus the CDT strategy), with both being more sensitive than the alcohol-oriented strategy using self-reported alcohol consumption.

Using the identified independent risk factors, the percentage of confirmed fibrosis detected among screened subjects increased to 5.2% (30/581) for males, to 9.4% (26/276) for males 60 years or older, to 16.4% (20/122) in males 60 years or older with CDT over 1.6%, and to 28.6% (6/21) in males 60 years or older with CDT over 1.6% and waist circumference 102 cm or greater. The corresponding number of subjects who would be required to undergo fibrosis screening in order to detect one advanced fibrosis was 19, 11, 6 and 4.

Subjects with possible underestimation of their alcohol consumption (CDT greater than 1.6% and low self report consumption represented 42% (30/72) of patients with presumed advanced fibrosis versus 21% (216/1022; P = 0.0001) of patients without fibrosis. Using CDT versus reported alcohol consumption, the attributable cause of liver fibrosis was ALD in 16% (12/74) versus 5% (4/74), mixed ALD/NAFLD in 45% (33/74) versus 23% (17/74), and NAFLD in 34% (25/74) versus 55% (41/74), respectively (P = 0.01).

### Discordance analysis between FT and LSM

A total of 871 subjects had an interpretable FT and LSM, 766 during screening and 105 during reinvestigation. During screening, the concordance between the 2 biomarkers was 91.2% (P = 0.009) (Table 5) and 99.1% (761/766 P < 0.0001) for the diagnosis of cirrhosis.

**Table 5:** Characteristics of naive subjects with and without discordance between FibroTest and LSM for the diagnosis of advanced fibrosis

Characteristics	Discordance		Significance			
	**No**	**Yes**	**Univariate**	**Multivariate**		

			**P**	**OR**	**95%CI**	**P**

Number of subjects	698	68				
Age at serum, years	57.0	59.7	0.04	1.02	1.00-1.06	0.048
Male (%)	349/698 (50%)	49/68 (72%)	0.0005	2.45	1.39-4.30	0.002
Thoracic fold	16.4	18.4	0.03	1.05	1.01-1.08	0.01
Operator effect	299/698 (43%)	40/68 (59%)	0.01	2.12	1.28-2.12	0.005
Cholesterol (mmol/L)	5.7	5.2	0.0002	0.58	0.44-0.76	<0.0001
Weight (kg)	69.4	73.2	0.04	Not	Included final model	NS
Waist circumference	83	87	0.005	Not	Included final model	NS
Body mass index	24.6	25.1	0.28	Not	Included final model	NS
ALT	24.7	28.2	0.007	Not	Included final model	NS
Fasting Glucose (mmol/L)	5.3	5.6	0.01	Not	Included final model	NS
HDL-cholesterol (mmol/L)	0.61	0.55	0.002	Not	Included final model	NS
Triglycerides (mmol/L)	1.2	1.2	0.81	Not	Included final model	NS
SteatoTest	0.31	0.36	0.04	Not	Included final model	NS

Using FT, 25 (3.3%) subjects had presumed advanced fibrosis versus 53 (6.9%) subjects using LSM. 48 (6.3%) subjects were suspected to be false negatives of FT or false positives of LSM, and 20 (2.6%) false negatives of LSM or false positives of FT. The 68 subjects with discordance had significantly more factors already identified as LSM variability factors than the other subjects. In multivariate analysis, male gender (OR = 2.45, 95%CI, 1.39-4.30; P = 0.002), total cholesterol (negatively correlated OR = 0.58, 95%CI, 0.44-0.76; P < 0.0001), thoracic fold (OR = 1.05, 95%CI, 1.01-1.08; P = 0.01), and one operator (OR = 2.12, 95%CI, 1.28-2.12; P = 0.005) were significantly associated with discordance.

### Estimate of FT false negative rate using LSM

Among the 48 (6.3%) subjects suspected to be false negatives of FT or false positives of LSM, 18 accepted to be reinvestigated (Table 6). Repeated FT results were consistently lower than 0.48. Repeated LSM in twelve subjects was no longer elevated and the false positivity of initial LSM was highly suspected; in 11 of these 12 subjects the initial LSM had been assessed by the "discordant" operator, and 4 had a thoracic fold over 15 mm. One case of a false negative FT was highly suspected, as the second LSM was still elevated (7.1 kPa) without any LSM variability risk factor. If the rate of false negative was the same in reinvestigated or not reinvestigated subjects, a total of 3 false negative FTs would have been expected among the 48 discordant cases, i.e., an overall false negative rate of 0.4% (3/766).

**Table 6:** Characteristics of discordant subjects at risk of false positive LSM or false negative FibroTest

Characteristics	Discordant not reinvestigated	Discordant reinvestigated
Number of subjects	30	18
Age >60 years	14/30 (47%)	4/18 (22%)
Male (%)	21 (70%)	12 (67%)
**Fatty liver risk factor (Alcohol or metabolic)**		
Self-declared alcohol consumption at risk*	8/30 (27%)	2/18 (11%)
Elevated Carbohydrate Deficient Transferin (>1.6%)	9/30 (30%)	12/18 (67%) (P = 0.01)
Alcohol at risk (either consumption or CDT)	16/30 (53%)	13/18 (72%)
BMI >= 27.0	10/30 (33%)	4/18 (22%)
***Metabolic factor of ATP-III classification (at least one)***		
Glucose >= 6.1 mmol/L or diabetes treatment	7/30 (23%)	2/18 (11%)
Central obesity waist >102 male >88 female	7/30 (23%)	2/18 (11%)
Triglycerides >= 1.7 mmol/L or fibrate treatment	8/30 (27%)	2/17 (12%)
Hypertension or treatment	12/30 (40%)	2/18 (11%) (P = 0.05)
HDL-cholesterol <1.03 mmol/L male <1.29 mmol/L female	2/30 (7%)	1/18 (6%)
Steatosis predicted by SteatoTest	7/30 (23%)	3/17 (18%)
HCV antibody positive	0/30 (0%)	0/18 (0%)
HIV antibody positive	0/30 (0%)	0/18 (0%)
HBsAg antigen positive	1/30 (3%)	0/18 (0%)

*More than one drink per day for female and more than two drinks per day for male

## Discussion

This study has limitations, but for the first time noninvasive biomarkers have been used to assess the prevalence of advanced liver fibrosis, to identify the independent associated risk factors and to prepare mass screening strategies. This study is a first step in order to assess the feasibility of using biomarkers, and not designed to assess the treatment and follow-up of the screen-detected patients [1]. The best method for validating a mass screening strategy is probably a large randomized trial [1]. The costs also were not estimated and compared. The diagnostic performance of FT is superior to other non-patented fibrosis biomarkers (i.e; APRI) but the cost is greater [4-6]. If accepted in screening strategies, the patented tests' price should be reduced according to the market volume and cost-benefit analyses.

### Benefit-risk of biomarkers

The present study is based on the assumption that FT and LSM have been sufficiently validated to be used as first-line screening and confirmation tests, respectively, for the diagnosis of advanced fibrosis, without the use of liver biopsy.

If these validations are accepted, biopsy is not necessary. It is because biomarkers have been previously validated that large screening studies are possible without the limitations of biopsy. To reduce the risk of false positive and false negative we used two validated biomarkers, the most sensitive being FT used as the screen test and elastography as a confirmatory test.

Up until recently, only liver biopsy was considered as a gold standard for the confirmation of suspected advanced fibrosis in subjects with abnormal standard liver tests [4]. Previous studies performed in community-based populations mostly used transaminases ALT as a first-line screening test [23-26]. This standard design is not accurate due to limitations of both standard liver tests and liver biopsy. Standard liver tests are significantly less accurate than FT for the diagnosis and prognosis of advanced fibrosis in patients with chronic hepatitis C, B, ALD, and for the diagnosis of fibrosis in patients with NAFLD [4-8]. In the present study only 33% of patients with confirmed fibrosis had ALT greater or equal to 50 IU/L.

In the present study biopsy was proposed to all the reinvestigated subjects with discordance between FT and LSM. 98% out of discordant subjects refused the biopsy, because of the risk of adverse events. In 2007 two deaths attributable to liver biopsy were reported to a French nationwide malpractice insurance company [27]. Besides these risks liver biopsy is not a perfect reference standard and even if all subjects with suspected fibrosis would have accepted liver biopsy as a confirmation test, there still would have been a risk of 25% for false positives/negatives after liver biopsy [28,29]. Several diagnostic and prospective prognostic studies have consistently demonstrated that in case of discordances between FT and biopsy, half of the cases were due to failure of biopsy [5,6].

Other patented or not fibrosis biomarkers, ELF, Hepascore and Fibrometer, NAFLD fibrosis score are potential candidates but have been less validated than FT for their diagnostic and prognostic values [4].

FT has limitations with a risk of false positives due to Gilbert's syndrome and hemolysis, as well a risk of false negatives due to acute inflammation [6]. As previously observed in blood donors and diabetics [12], the high-risk profiles were rare (0.4%) in the present study with 99.6% applicability. Among applicable subjects the estimated rate of false negatives was 0.4% and the false positive rate ranged according to fibrosis definition from 0.08% to 1.4%.

LSM has been validated as an alternative to liver biopsy for the diagnosis of advanced fibrosis in the most frequent liver diseases [9,30]. Compared to FT, the main disadvantages of LSM for a first-line test are the lower applicability rate, the higher number of variability factors including a possible operator effect and the lower sensitivity for earlier stages of fibrosis [10].

In the present analysis we focus on confirmed fibrosis, which is a minimal hypothesis in term of efficacy. The fact that 50% of patients with presumed advanced fibrosis have not been confirmed by LSM can be viewed as a weakness of the present study. These individuals declined a biopsy and they could be false positive of FT or false negative of LSM. We acknowledge that the strategy combining FT as a first-line screening test and LSM as a confirmation test magnified the complimentary advantages of these two biomarkers for F3 and F4 but not for F2. We previously observed a lower sensitivity of LSM versus FT, for early stage F2 in patients with NAFLD, but this point must be confirmed by other studies [11]. If the higher accuracy of FT for the diagnosis of F2 is confirmed the screening efficacy would be doubled.

### Prevalence of advanced liver fibrosis

Depending on the definitions, and on bias related to the non reinvestigated population, the estimated prevalence of advanced fibrosis varied from 0.7% to 2.8%. However the lower estimated prevalence of 0.7% seems not realistic as the non reinvestigated population was similar to the reinvestigated population for the main characteristics (Table 1).

Using standard liver tests, a higher prevalence of "chronic liver disease" was observed in Italy, the USA and China, from 7.9% to 17.5% of estimates [23-26,31]. Without specific markers of advanced fibrosis, the attributable cause of abnormal liver tests could include non severe liver disease that is steatosis, or inflammation without advanced fibrosis.

In the present study the most common attributable cause of fibrosis was NAFLD associated with ALD or alone (77%), much higher than hepatitis C (8%) and ALD alone (8%). Among subjects with confirmed cirrhosis, HCV infection was more prevalent (22%), and was always associated with alcohol consumption. This study was not designed to estimate the possible role of drugs in inducing liver injury.

Other studies in the general population have also found NAFLD to be the leading cause of suspected chronic liver disease [23-26]. In the present study the prevalence of steatosis assessed using SteatoTest was expected, 18% for moderate to severe grade and 23% for minimal steatosis, as well as 1% for NASH.

One original observation of the present study was that the use of CDT, a biomarker of excessive alcohol consumption, instead of self-reported alcohol consumption, permitted to attribute fibrosis to ALD 3 times more often and to mixed ALD/NAFLD 2 times more often. One limitation of the present study was that CDT was assessed in a subpopulation of 1097 consecutive subjects and these results need to be replicated in a larger population. The cost of CDT is also a limitation for large screening strategies.

### Risk factors

The results confirmed that age, male gender and HBsAg or HCV antibodies were associated with fibrosis. The original observation was that waist circumference was the best independent predictive factor for fibrosis among the "metabolic factors", when CDT was used as a biomarker of alcohol consumption. The significant association between triglycerides and fibrosis was no longer significant after adjustment using CDT. This was expected, as triglycerides are associated both with metabolic factors and alcohol consumption; finally, this observation also supports a lack of sensitivity of self-reported alcohol consumption.

### Screening strategies

The results suggest that the non selective strategy using FT as a first-line test in the social security health centers and elastography in the reference center seems feasible and effective.

According to official recommendations, so far only screening for alcohol misuse in adults, and lipid disorder in men aged 35 and older are recommended in general population. Screening for hepatitis B or C or hemochromatosis in general population are not recommended [1,32]. For primary liver cancer two randomized trials have suggested the efficacy of cancer screening in chronic carriers of hepatitis B virus [33,34]. In the present study the standard "hepatitis oriented strategy" was less effective than a non selective strategy.

### Advantages and limitations of the non selective strategy

The advantage of the present screening was a simple definition of target population and individuals: subjects volunteers affiliated to the national social security system (covering 90% of French people), of 40 years or older consulting in health centers. Another advantage is the acceptance rate of FT in this population (99.8%) and the high rate of interpretable FT (99.6%).

A mean of 144 subjects would be required to undergo fibrosis screening in order to detect one advanced fibrosis case. This effectiveness is comparable to published strategies screening for advanced colon neoplasia [35].

Only 50% of subjects with presumed advanced fibrosis have been re-investigated (compliance with elastography recommendation) in clinical facilities, despite two letters. This rate is the usual magnitude of screening strategies for colon cancer but lower than that observed for prostate cancer screening (86% compliance with biopsy recommendation) [36]. This point should be improved.

### Effectiveness and limitations of selective strategies

The usual selective strategy used in the health centers, oriented to hepatitis C and B high-risk subjects, was significantly less effective due to a lack of sensitivity. This is in accordance with other areas such as cancer screening [1].

Using four simple identified independent factors (gender, age, alcohol consumption estimated with CDT, and waist circumference), the number of subjects required to undergo fibrosis screening in order to detect one advanced fibrosis case already decreased from 149 to 11 for men aged 60 years or older. This is comparable to the number of men required to undergo colonoscopy screening in order to detect one advanced case of neoplasia, which ranged from 23 to 10 according to age [35]. However this could be different for a strategy designed for cirrhosis screening as metabolic factors are less associated so far with cirrhosis than chronic viral hepatitis.

## Conclusions

Non invasive biomarker FibroTest has permitted to estimate prevalence of advanced fibrosis around 2.8% in a French general population aged 40 years or older. It has also permitted to identify several independent risk factors which may be used for the validation of selective or non-selective screening strategies.

These results suggest to organize a large randomized trial in order to estimate the impact of fibrosis screening on liver related complications and mortality.

## Competing interests

Thierry Poynard is the inventor and has a capital interest in Biopredictive, the company marketing FibroTest. Mona Munteanu and Fabienne Drane are employee of Biopredictive. The patents belong to the public organization Assistance Publique Hôpitaux de Paris.

## Authors' contributions

TP has conceived the study, analyzed the data and has written the article. PL, PI, AV, YN, PN, MM, JM, VR have included patients and performed elastography. MM and FD checked the interpretability of biomarkers according to the manufacturer recommendations. DM, FIB, and JPC have organized and performed biomarkers analyses. BV and JPG organized the screening in the Social Security health examination centers. All authors read and approved the final manuscript.

## Pre-publication history

The pre-publication history for this paper can be accessed here:

http://www.biomedcentral.com/1471-230X/10/40/prepub

## Supplementary Material

Additional file 1 **Characteristics of included subjects in comparison with French population**. A table including the main characteristics of included subjects which were similar to those of the French population. Click here for file

Additional file 2 **Attributable cause of cirrhosis in the population without history of liver disease**. A table describing the main characteristics of subjects with presumed cirrhosis Click here for file

Additional file 3 **Predictive values of oriented screening strategies in CDT populations**. A table describing the predictive values of alcohol-oriented screening strategies in populations who had a consecutive assay of Carbohydrate Deficient Transferrin Click here for file

## References

[B1] Hakama M Coleman MP Alexe DM Auvinen A Cancer screening: evidence and practice in Europe 2008 Eur J Cancer 2008 44 1404 13 10.1016/j.ejca.2008.02.013 18343653

[B2] World Health Organization Revised global burden of disease 2002 estimates http://www.who.int/healthinfo/global_burden_disease/en/index.html Accessed October 27th 2008

[B3] Poynard T Mathurin P Lai CL Guyader D Poupon R Tainturier MH Myers RP Muntenau M Ratziu V Manns M Vogel A Capron F Chedid A Bedossa P A comparison of fibrosis progression in chronic liver diseases J Hepatol 2003 38 257 65 10.1016/S0168-8278(02)00413-0 12586290

[B4] Manning DS Afdhal NH Diagnosis and quantitation of fibrosis Gastroenterology 2008 134 1670 81 10.1053/j.gastro.2008.03.001 18471546

[B5] Poynard T Muntenau M Morra R Ngo Y Imbert-Bismut F Thabut D Messous D Massard J Lebray P Moussalli J Benhamou Y Ratziu V Methodological aspects for the interpretation of liver fibrosis non-invasive biomarkers: a 2008 update Gastroenterol Clin Biol 2008 32 8 21 18973843 10.1016/S0399-8320(08)73990-3

[B6] Halfon P Munteanu M Poynard T FibroTest-ActiTest as a non-invasive marker of liver ?brosis Gastroenterol Clin Biol 2008 32 22 38 18973844 10.1016/S0399-8320(08)73991-5

[B7] Ngo Y Benhamou Y Thibault V Ingiliz P Munteanu M Lebray P Thabut D Morra R Messous D Charlotte F Imbert-Bismut F Bonnefont-Rousselot D Moussalli J Ratziu V Poynard T An accurate definition of the status of inactive hepatitis B virus carrier by a combination of biomarkers (Fibrotest-Actitest) and viral load PlosOne 2008 3 e2573 10.1371/journal.pone.0002573 PMC2440801 18596917

[B8] Naveau S Gaudé G Asnacios A Agostini H Abella A Barri-Ova N Dauvois B Prévot S Ngo Y Munteanu M Balian A Njiké-Nakseu M Perlemuter G Poynard T Diagnostic and prognostic values of non-invasive biomarkers of fibrosis in patients with alcoholic liver disease Hepatology 2009 49 97 105 10.1002/hep.22576 19053048

[B9] Friedrich-Rust M Ong MF Martens S Sarrazin C Bojunga J Zeuzem S Herrmann E Performance of transient elastography for the staging of liver fibrosis: a meta-analysis Gastroenterology 2008 134 960 74 10.1053/j.gastro.2008.01.034 18395077

[B10] Poynard T Ingiliz P Elkrief L Munteanu M Lebray P Morra R Messous D Bismut FI Roulot D Benhamou Y Thabut D Ratziu V Concordance in a world without a gold standard: A new non-invasive methodology for improving accuracy of fibrosis markers PlosOne 2008 3 e3857 10.1371/journal.pone.0003857 PMC2586659 19052646

[B11] Ratziu V Giral P Munteanu M Messous D Mercadier A Bernard M Morra R Imbert-Bismut F Bruckert E Poynard T Screening for liver disease using non-invasive biomarkers (FibroTest-SteatoTest-NashTest-FibroSURE) in patients with hyperlipidaemia Aliment Pharmacol Ther 2007 25 207 18 17229244 10.1111/j.1365-2036.2006.03182.x

[B12] Jacqueminet S Lebray P Morra R Munteanu M Devers L Messous D Bernard M Hartemann-Heurtier A Imbert-Bismut F Ratziu V Grimaldi A Poynard T Screening for liver fibrosis by using a noninvasive biomarker in patients with diabetes Clin Gastroenterol Hepatol 2008 6 828 31 10.1016/j.cgh.2008.03.005 18524692

[B13] Friedman SL Bansal MB Reversal of hepatic fibrosis -- fact or fantasy? Hepatology 2006 43 2 Suppl 1 S82 8 10.1002/hep.20974 16447275

[B14] Poynard T Calès P Pasta L Ideo G Pascal JP Pagliaro L Lebrec D Beta-adrenergic-antagonist drugs in the prevention of gastrointestinal bleeding in patients with cirrhosis and esophageal varices N Engl J Med 1991 30 324 1532 8 10.1056/NEJM199105303242202 1674104

[B15] Imbert-Bismut F Naveau S Morra R Munteanu M Ratziu V Abella A Messous D Thabut D Benhamou Y Poynard T The diagnostic value of combining carbohydrate deficient transferin, fibrosis and steatosis biomarkers for the prediction of excessive alcohol consumption Eur J Gastroenterol Hepatol 2009 21 18 27 10.1097/MEG.0b013e32830a4f4c 19011575

[B16] Becker U Deis A Sørensen TI Grønbaek M Borch-Johnsen K Müller CF Schnohr P Jensen G Prediction of risk of liver disease by alcohol intake, sex, and age: a prospective population study Hepatology 1996 23 1025 9 10.1002/hep.510230513 8621128

[B17] Castéra L Vergniol J Foucher J Le Bail B Chanteloup E Haaser M Darriet M Couzigou P De Lédinghen V Prospective comparison of transient elastography, Fibrotest, APRI and liver biopsy for the assessment of fibrosis in chronic hepatitis C Gastroenterology 2005 128 343 50 10.1053/j.gastro.2004.11.018 15685546

[B18] Castéra L Foucher J Bernard PH Carvalho F Allaix D Merrouche W Couzigou P de Lédinghen V Pitfalls of liver stiffness measurement: A 5-year prospective study of 13,369 examinations Hepatology 2010 51 828 35 20063276 10.1002/hep.23425

[B19] Roulot D Czernichow S Le Clésiau H Costes JL Vergnaud AC Beaugrand M Liver stiffness values in apparently healthy subjects: Influence of gender and metabolic syndrome J Hepatol 2008 48 606 613 10.1016/S0168-8278(08)60967-8 18222014

[B20] Institut de Veille Sanitaire Prevalence of hepatitis B and hepatitis C in France http://www.invs.sante.fr/publications/2006/prevalence_b_c/index.html Accessed November 16th 2008

[B21] French national survey on nutrition and health, ENNS http://www.invs.sante.fr/publications/2007/nutrition_enns/index.html Accessed November 16th 2008

[B22] Hintze JL NCSS 2007 User Guide Number Cruncher Statistical Systems software NCSS, Kaysville, Utah 2007

[B23] Bedogni G Miglioli L Masutti F Tiribelli C Marchesini G Bellentani S Prevalence of and risk factors for nonalcoholic fatty liver disease: the Dionysos nutrition and liver study Hepatology 2005 42 44 52 10.1002/hep.20734 15895401

[B24] Clark JM Brancati FL Diehl AM The prevalence and etiology of elevated aminotransferase levels in the United States Am J Gastroenterol 2003 98 960 7 10.1111/j.1572-0241.2003.07486.x 12809815

[B25] Chen CH Huang MH Yang JC Nien CK Yang CC Yeh YH Yueh SK Prevalence and etiology of elevated serum alanine aminotransferase level in an adult population in Taiwan J Gastroenterol Hepatol 2007 22 1482 9 10.1111/j.1440-1746.2006.04615.x 17716352

[B26] Pendino GM Mariano A Surace P Caserta CA Fiorillo MT Amante A Bruno S Mangano C Polito I Amato F Cotichini R Stroffolini T Mele A ACE Collaborating Group Prevalence and etiology of altered liver tests: a population-based survey in a Mediterranean town Hepatology 2005 41 1151 9 10.1002/hep.20689 15841464

[B27] Poynard T Benhamou Y Thabut D Ratziu V Liver Biopsy: the best standard... when everything else fails J Hepatol 2009 50 850 8 10.1016/j.jhep.2009.02.010 19380171

[B28] Bedossa P Dargère D Paradis V Sampling variability of liver fibrosis in chronic hepatitis C Hepatology 2003 38 1449 1457 14647056 10.1016/j.hep.2003.09.022

[B29] Ratziu V Charlotte F Heurtier A Gombert S Giral P Bruckert E Grimaldi A Capron F Poynard T LIDO Study Group Sampling variability of liver biopsy in nonalcoholic fatty liver disease Gastroenterology 2005 128 1898 1906 10.1053/j.gastro.2005.03.084 15940625

[B30] Yoneda M Yoneda M Mawatari H Fujita K Endo H Iida H Nozaki Y Yonemitsu K Higurashi T Takahashi H Kobayashi N Kirikoshi H Abe Y Inamori M Kubota K Saito S Tamano M Hiraishi H Maeyama S Yamaguchi N Togo S Nakajima A Noninvasive assessment of liver fibrosis by measurement of stiffness in patients with nonalcoholic fatty liver disease (NAFLD) Dig Liver Dis 2008 40 371 8 10.1016/j.dld.2007.10.019 18083083

[B31] Bell BP Manos MM Zaman A Terrault N Thomas A Navarro VJ Dhotre KB Murphy RC Van Ness GR Stabach N Robert ME Bower WA Bialek SR Sofair AN The epidemiology of newly diagnosed chronic liver disease in Gastroenterology. Practices in the united states: results from population-based surveillance Am J Gastroenterol 2008 103 2727 36 10.1111/j.1572-0241.2008.02071.x 18684170

[B32] U.S. Preventive Services Task Force Guide to clinical preventive services http://www.ahrq.gov/clinic/uspstf/uspstopics.htm Accessed April 2010

[B33] Chen JG Parkin DM Chen QG Screening for liver cancer: results of a randomised, controlled trial in Qidong, China J Med Screen 2003 10 204 9 10.1258/096914103771773320 14738659

[B34] Chen JG Parkin DM Chen QG Lu JH Shen QJ Zhang BC Zhu YR Randomized controlled trial of screening for hepatocellular carcinoma J Cancer Res Clin Oncol 2004 131 417 22 10.1007/s00432-004-0552-0 PMC12161851 15042359

[B35] Regula J Rupinski M Kraszewska E Polkowski M Pachlewski J Orlowska J Nowacki MP Butruk E Colonoscopy in colorectal-cancer screening for detection of advanced neoplasia N Engl J Med 2006 355 1863 72 10.1056/NEJMoa054967 17079760

[B36] Schröder FH Hugosson J Roobol MJ Tammela TL Ciatto S Nelen V Kwiatkowski M Lujan M Lilja H Zappa M Denis LJ Recker F Berenguer A Määttänen L Bangma CH Aus G Villers A Rebillard X Kwast T van der Blijenberg BG Moss SM de Koning HJ Auvinen A ERSPC Investigators Screening and prostate-cancer mortality in a randomized european study N Engl J Med 2009 360 1320 8 10.1056/NEJMoa0810084 19297566

