# Supplementary material for: Prevalence of liver fibrosis and risk factors in a general population using non-invasive biomarkers (FibroTest)
Source: BMC Gastroenterol. 2010 Apr 22;10:40. doi: 10.1186/1471-230X-10-40 (PMC2864202; doi:10.1186/1471-230X-10-40)
Supplement: Additional file 1 — Characteristics of included subjects in comparison with French population. A table including the main characteristics of included subjects which were similar to those of the French population. [file 1471-230X-10-40-S1.DOC]

**Additional File 1: Characteristics of included subjects in comparison with French population.**

| **Characteristics** | **Sample Included** | **Estimates for French population 18-80 years of age in millions** |
| --- | --- | --- |
| **Number of subjects** | **7463** | **43.4 (25,5 between 40 to 80 years)** |
| Male | 4113 (55%) | 21 (49%) |
| Female | 3350 (45%) | 22 (51%) |
| **Age** |  |  |
| 40-59 years | 4152 (56%) | 15,6 (57%) |
| 60-80 years | 3311 (44%) | 10.9 (43%) |
| **Alcohol consumption (self declaration)** |  |  |
| ***Male*** |  |  |
| No alcohol | 707 (17%) | 4.7 (22%) |
| 1-28 drinks per week | 3093 (75%) | 14.9 (71%) |
| More than 28 drinks per week | 308 (8%) | 1.4 (7%) |
| ***Female*** |  |  |
| No alcohol | 987 (30%) | 9.7 (44%) |
| 1-21 drinks per week | 2279 (68%) | 12.3 (55%) |
| More than 21 drinks per week | 82 (2%) | 0.2 (1%) |
| **Professional category** |  |  |
| Workman | 498 (7%) | 7.1 (19%) |
| Employee | 1813 (24%) | 16.3 (44%) |
| Intermediate | 2245 (30%) | 5.3 (14%) |
| High | 1969 (26%) | 7.0 (19%) |
| Other | 931 (12%) | 1.7 (4%) |
| **Professional status** |  |  |
| Active | 3903 (51%) | 24.3 (56%) |
| Retired | 2224 (31%) | 10.0 (23%) |
| Jobless | 1068 (14%) | 3.5 (8%) |
| Spouse or other | 261 (4%) | 5.4 (13%) |
| **Education level** |  |  |
| Low | 2999 (41%) | 23.4 (54%) |
| Intermediate | 2220 (31%) | 13.1 (31%) |
| High | 2028 (28%) | 6.3 (15%) |
| **Residency** |  |  |
| Paris area | 7463 (100%) | 8.2 (19%) |
| Other | 0 (0%) | 35.2 (81%) |
| **Region origin** |  |  |
| Caucasian | 6678 (90%) | 39,7 (91%) |
| North Africa | 465 (6%) | 2.2 (5%) |
| Other Africa | 226 (3%) | 1.1 (3%) |
| Asia | 93 (1%) | 0.4 (1%) |
| **HCV antibody positive** | 32/3260 (0.89%) | 0.37 (0.84%) |
| **HBsAg positive** | 8/717 (1.12%) | 0.28 (0.65%) |
