# Supplementary material for: Prevalence of liver fibrosis and risk factors in a general population using non-invasive biomarkers (FibroTest)
Source: BMC Gastroenterol. 2010 Apr 22;10:40. doi: 10.1186/1471-230X-10-40 (PMC2864202; doi:10.1186/1471-230X-10-40)
Supplement: Additional file 2 — Attributable cause of cirrhosis in the population without history of liver disease. A table describing the main characteristics of subjects with presumed cirrhosis [file 1471-230X-10-40-S2.DOC]

**Additional File 2: Attributable cause of cirrhosis in the population without history of liver disease**

| **Characteristics** | **All** | **Reinvestigated** | | | | **Not reinvestigated** |
| --- | --- | --- | --- | --- | --- | --- |
|  | **Presumed cirrhosis** | **Cirrhosis Confirmed** | **Cirrhosis Highly suspected** | **Fibrosis still suspected** | **All reinvestigated** |  |
| Number of subjects | 25 | 9 | 1 | 1 | 11 | 14 |
| ***Cause of liver disease among cirrhosis including CDT or self declaration if CDT not performed*** |  |  |  |  |  |  |
| Non alcoholic fatty liver disease* | 14 (56%) | 3 (33%) | 1 (100%) | 1(100%) | 5 (39%) | 9 (64%) |
| Alcoholic liver disease** | 0 (0%) | 0 (0%) | 0 (0%) | 0 (0%) | 0 (0%) | 0 (0%) |
| Non alcoholic and alcoholic | 7 (28%) | 4 (44%) | 0 (0%) | 0 (0%) | 4 (36%) | 3 (21%) |
| Chronic hepatitis C | 3 (12%) | 2 (22%) | 0 (0%) | 0 (0%) | 2 (18%) | 1 (7%) |
| Chronic hepatitis B | 0 (0%) | 0 (0%) | 0 (0%) | 0 (0%) | 0 (0%) | 0 (0%) |
| Hemochromatosis | 0 (0%) | 0 (0%) | 0 (0%) | 0 (0%) | 0 (0%) | 0 (0%) |
| Auto-immune hepatitis | 0 (0%) | 0 (0%) | 0 (0%) | 0 (0%) | 0 (0%) | 0 (0%) |
| No risk factor | 1 (4%) | 0 (0%) | 0 (0%) | 0 (0%) | 0 (9%) | 1 (7%) |

* At least one factor of the metabolic syndrome without alcohol consumption at risk

**Alcohol consumption at risk self declared or CDT >1.6% without metabolic factor
