# Supplementary material for: Prevalence of liver fibrosis and risk factors in a general population using non-invasive biomarkers (FibroTest)
Source: BMC Gastroenterol. 2010 Apr 22;10:40. doi: 10.1186/1471-230X-10-40 (PMC2864202; doi:10.1186/1471-230X-10-40)
Supplement: Additional file 3 — Predictive values of oriented screening strategies in CDT populations. A table describing the predictive values of alcohol-oriented screening strategies in populations who had a consecutive assay of Carbohydrate Deficient Transferrin [file 1471-230X-10-40-S3.DOC]

**Additional File 3: Predictive values of oriented screening strategies in CDT populations**

| **Strategy** | **Number subjects** | **Presumed fibrosis** | **Confirmed fibrosis** |
| --- | --- | --- | --- |
| ***Reported consumption-oriented in CDT population*** |  |  |  |
| Predictive value |  |  |  |
| >10g female 20g male | 230 | 21 (9.1%) | 12 (5.2%) |
| <=10g female 20g male | 867 | 53 (6.1%) | 18 (2.1%) |
| Odds Ratio | 1097 | 1.5 (0.9-2.7) | 2.7 (1.2-5.8) |
| Area under ROC curve | 1097 | 0.58(0.51-0.64) | 0.60 (0.48-0.70) |
| ***Metabolic factors-oriented in CDT population*** |  |  |  |
| Predictive value |  |  |  |
| At least one metabolic factor | 594 | 58 (9.8%) | 23 (3.9%) |
| None | 503 | 16 (3.2%) | 7 (1.4%) |
| Odds ratio | 1097 | 3.3 (1.8-6.1) | 3.1 (1.3-8.0) |
